# Biosynthesis of ZnO Nanoparticles by a New *Pichia kudriavzevii* Yeast Strain and Evaluation of Their Antimicrobial and Antioxidant Activities

**DOI:** 10.3390/molecules22060872

**Published:** 2017-05-24

**Authors:** Amin Boroumand Moghaddam, Mona Moniri, Susan Azizi, Raha Abdul Rahim, Arbakariya Bin Ariff, Wan Zuhainis Saad, Farideh Namvar, Mohammad Navaderi, Rosfarizan Mohamad

**Affiliations:** 1 Department of Bioprocess Technology, Faculty of Biotechnology and Biomolecular Sciences, Universiti Putra Malaysia, 43400 UPM Serdang, Selangor, Malaysia; amin.broomandm@yahoo.com (A.B.M.); mona_moniri6@yahoo.com (M.M.); arbarif@upm.edu.my (A.B.A.); 2 Young Research and Elite Club, Sabzevar Branch, Islamic Azad University, Sabzevar, Iran; 3 Department of Cell and Molecular Biology, Faculty of Biotechnology and Biomolecular Sciences, Universiti Putra Malaysia, 43400 UPM Serdang, Selangor, Malaysia; raha@upm.edu.my; 4 Bioprocessing and Biomanufacturing Research Centre, Faculty of Biotechnology and Biomolecular Sciences, Universiti Putra Malaysia, 43400 UPM Serdang, Selangor, Malaysia; 5 Department of Microbiology, Faculty of Biotechnology and Biomolecular Sciences, Universiti Putra Malaysia, 43400 UPM Serdang, Selangor, Malaysia; zuhainis@upm.edu.my; 6 Institute of Tropical Forestry and Forest Products, Universiti Putra Malaysia, 43400 UPM Serdang, Selangor, Malaysia; 7 Department of Medicine, Mashhad Branch, Islamic Azad University, Mashhad, Iran; farideh.namvar@gmail.com; 8 Young Research and Elite Club, Parand Branch, Islamic Azad University, Parand, Iran; navaderimohammad@yahoo.com

**Keywords:** green synthesis, zinc oxide nanoparticles, *Pichia kudriavzevii*, antibacterial activity, antioxidant

## Abstract

The potential ability of a new yeast strain, *Pichia kudriavzevii*, in the synthesis of zinc oxide nanoparticles (ZnO-NPs) through a green method was explored in this study. The effect of reaction time (12, 24 and 36 h) on the structure of the resulting ZnO nanoparticles was investigated. From the XRD and TEM results, the ZnO-NPs with a hexagonal wurtzite structure and a particle crystal size of ~10–61 nm was formed at different reaction times. Combing XRD, TEM, and PL results, it was revealed that the sample prepared at intermediate duration (24 h) has the most favorable nanosized structure with the lowest defect concentration. The biomedical properties of ZnO-NPs as free radical scavenging activity, cytotoxicity and antibacterial agents were characterized. Biosynthesized ZnO-NPs showed strong DPPH free radical scavenging and a dose dependent toxicity with non-toxic effects on Vero cells for concentrations below 190 µg/mL. Desirable bactericidal activity was shown by the ZnO-NPs on Gram-positive bacteria (*Bacillus subtilis*, *Staphylococcus epidermidis* and *Staphylococcus aurous*) and Gram-negative bacteria (*Escherichia coli* and *Serratia marcescens*). A maximum inhibition zone of ~19 mm was observed for *Staphylococcus epidermidis* at a concentration of 100 µg/mL for sample prepared at 24 h. The results from this study reveal that ZnO-NPs possesses potential for many medical and industrial applications.

## 1. Introduction

Nanotechnology provides many possibilities in various scientific and technological fields [1]. Researchers are becoming increasingly interested in pharmacological nanotechnology due to its widespread applications. Nanoparticles can be produced by either chemical, physical and biotic processes [2]. In the last two decades, biosynthesis of NPs has received considerable attention due to the growing need to develop environmentally benign technologies in material synthesis [3]. For instance, biotic synthesis of metal NPs uses microorganisms such as fungi [4], yeast [5], bacteria [6,7], plants [8] and algae [9].

The potential use of yeasts in NP biosynthesis is promising because of the ease of handling yeasts under laboratory conditions, their abundant enzyme synthesis and rapid growth without the need of complex nutrients [10,11]. Economic longevity and capacity for employing biomass is yet another advantage of using the green approach employing yeasts to produce metallic NPs. Furthermore, numerous yeasts species grow at accelerated rates and therefore producing cultures as well as their storage in the laboratory is very practical [12]. Yeast biomasses are capable of producing metal nanoparticles and nanostructures through the reduction of proteins in enzymes either intracellularly or extracellularly [13]. The reductive properties of proteins are related to many functional groups such as –NH_2_, –OH, C=O, C–O–C, C–N–C, and –S–S–, which enable the donation of electrons to form NP from metal ions.

ZnO, a direct wide band-gap (3.2 eV) semiconductor, has been used for a wide range of applications, including those involved with ultraviolet radiation [14], optoelectronics [15,16] and biological sciences [17,18]. In crystalline ZnO, when the irradiation has a greater energy than the band-gap energy of ZnO, the electrons in the valence band (VB) will be excited to the conduction band (CB), and thus form electron–hole pairs. These electrons and holes often quickly recombine, but can also move to the NP surface and react with species that are adsorbed there, thus enabling the reaction of electrons with oxygen and holes (h^−^) with water or hydroxyl ions to form hydroxyl and superoxide radicals. This property of ZnO has been used for photocatalytic oxidation of pollutants as well as sensitizers for bacteria [19] and cancer cell photodestruction via oxidative damage [20,21]. Since conventional chemical and physical approaches are generally expensive and require toxic chemical compounds as a reaction medium, the synthesis of ZnO-NPs using “green” processes has merited substantial attention [22]. Green synthesis is an eco-friendly method for the production of nontoxic and biocompatible nanoparticles. The chemical components in biomass materials are acting as reducing agents and also as the stabilizing agents by attaching on the surface of the NPs formed, thus preventing their aggregation and controlling the particle size. The biosynthesized ZnO-NPs are suitable to be used as drug carriers, cosmetics, and fillings in medicinal materials [23].

The objective of this present study was to design a green approach to synthesize ZnO-NPs using a newly isolated yeast strain, *Pichia kudriavzevii* (*P. kudriavzevii*). *Pichia kudriavzevii* is synonymously known as *Issatchenkia orientalis* and is an anamorph of *Candida krusei* [24,25]. This yeast strain has been isolated from food and fruit sources, such as sourdoughs [26], fermented butter-like products [27] and the starter culture of Tanzanian fermented togwa [28]. The effect of reaction time on structure, morphology and size of the as-synthesized nanoparticles was evaluated. Furthermore, free radical scavenging activity, cytotoxicity and in vitro antimicrobial activities of ZnO-NPs against various gram-negative and gram-positive bacteria were also investigated.

## 2. Results and Discussion

**Figure 1:**
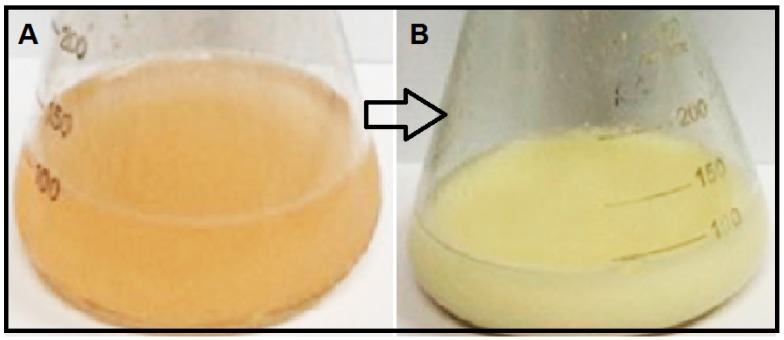
The changes in color of Zn^2+^/*P. kudriavzevii* suspension (**A**) at 0 h; and (**B**) 24 h of incubation.

Green synthesis of metal oxide nanoparticles using biomass materials is attractive because these approaches are simple, low-cost and ecofriendly as compared to chemical and physical methods. The present study describes the extracellular synthesis of ZnO-NPs via *P. kudriavzevii*. The initial formation of ZnO was visually observed through the changes in color of Zn^2+^/biomass suspensions from light brown to cream color after the incubation for 12, 24 and 36 h. A deep white color was observed after 24 h of incubation and remained steady until 36 h (Figure 1). The biochemical mechanism of nanoparticle formation and stabilization still remains largely unexplored, but some research groups have demonstrated that the proteins present in enzymes secreted by microorganisms are the principal biomolecules involved in the formation of metal/metal oxide nanoparticles [29,30]. Hydroxyl groups of amino acids are the most active functional groups which allow the complexation of Zn ions to these molecules follows with hydrolysis, and finally the synthesis of ZnO nanoparticles through thermal decomposition. This structure assists amino acids to stabilize zinc particles and finally ZnO-NPs while inhibiting their extreme aggregation or crystal growth.

### 2.1. Characterization of ZnO-NPs

#### 2.1.1. X-ray Diffraction Pattern (XRD)

**Figure 2:**
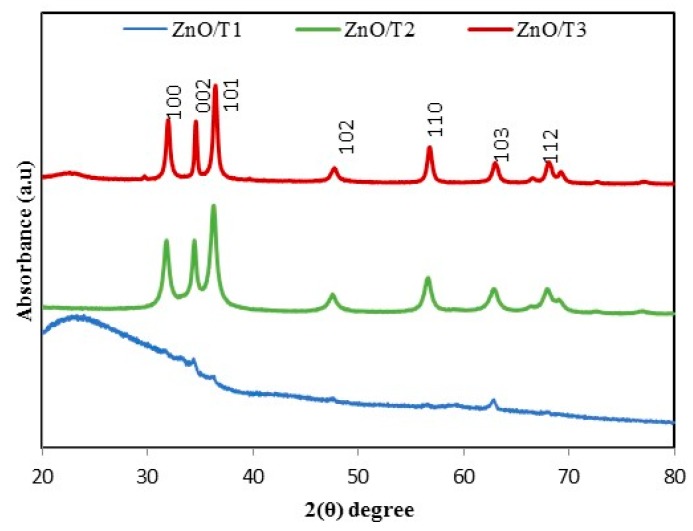
XRD pattern of biosynthesized ZnO-NPs at different reaction times, ZnO/T1 (12 h), ZnO/T2 (24 h) and ZnO/T3 (36 h).

The typical XRD patterns of ZnO nanoparticles synthesized by *P. kudriavzevii* at different reaction times are shown in Figure 2. All the peaks, (100), (002), (101), (102), (110), (103) and (112), presence in the XRD patterns of ZnO/T2 and ZnO/T3 samples can be well indexed to the hexagonal wurtzite structure (JCPDS card No. 01-079-0207). Strong intensity and widening of ZnO diffraction peaks indicate that the resulting product was highly crystalline in nature and the crystalline sizes of the obtained particles were in nanoscale range. On the other hand, only some of the diffraction peaks with low intensities were observed in ZnO/T1 sample, indicating that the obtained ZnO nanoparticles has low crystallinity with a wurtzite structure [31]. Average crystallite size was calculated using Scherrer’s formula, D $=\frac{0.9\lambda }{\beta \cos\theta }$, according to (101) diffraction peak, where D, λ, θ, and β indicate the average particle size, the X-ray wavelength (1.5406 Å), the Bragg diffraction angle and the full width at half maximum (FWHM) in radians, respectively. The particle size of the ZnO-NPs was 11.25, 38.48 and 54.27 nm for ZnO/T1, ZnO/T2 and ZnO/T3 samples, respectively.

#### 2.1.2. TEM Analysis

**Figure 3:**
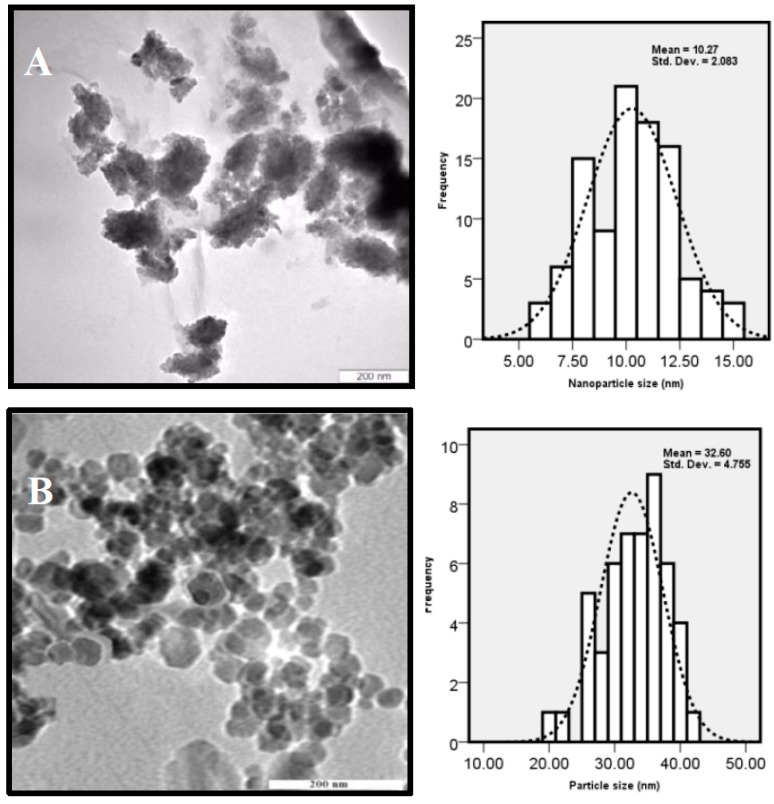

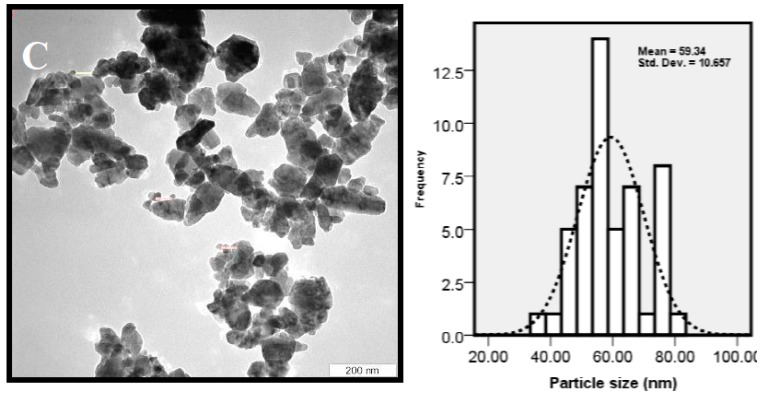
TEM micrograph of ZnO nanoparticles synthesized by *P. kudriavzevii* at various incubation times (**A**) 12; (**B**) 24 and (**C**) 36 h.

The size, shape, and distribution of as-synthesized ZnO-NPs were evaluated through TEM observations. Figure 3 shows the TEM images of ZnO NPs, which were prepared at 12, 24 and 36 h of incubation time. The results clearly show that the shape and crystal structure of nanoparticles were considerably developed when the reaction time was prolonged. The increase in crystal sizes of nanoparticles can be explained with the slow rate of formation of nanoparticles in the reaction medium [32]. Chemical reaction rate affects both the nucleation and growth processes. The nucleation and growth steps take place simultaneously when the chemical reaction is slow [33]. A slow chemical reaction favors a steady nuclei creation, in which, a certain number of nuclei in the system was maintained. Consequently, development of growth may be happened during the whole process, which explains that the larger particle size was gained with a longer incubation time.

The agglomerated and low crystallinity particles with a mean size of 10 ± 2.08 nm were synthesized at 12 h (Figure 3A), and subsequently were developed to the poly dispersed nanostructures with high crystallinity and hexagonal shape with an average particle size of 32 ± 4.7 nm at 24 h (Figure 3B). Some irregular shape and agglomerated nanoparticles with a mean size of 59 ± 10.6 nm were formed with prolonged incubation time (36 h) (Figure 3C). It is observed that the ZnO-NPs mostly tended to agglomeration due to high surface energy that generally happens when synthesis is performed in aqueous medium and also possibly owing to densification resulting in narrow space between particles. The XRD and TEM results showed that the favorable size, morphology and distribution were obtained for nanoparticles prepared with the incubation time of about 24 h.

#### 2.1.3. UV-Visible Spectrophotometry

**Figure 4:**
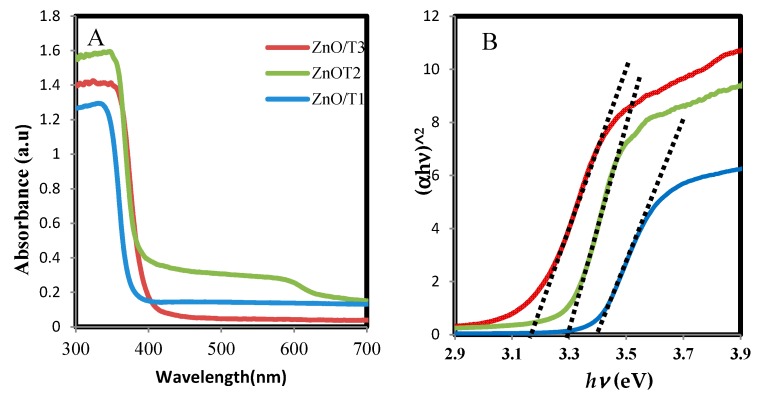
(**A**) Absorption spectra and (**B**) band gap of the ZnO-NPs prepared at different reaction times.

The UV-vis absorption spectra of biosynthesized ZnO-NPs samples are shown in Figure 4A. The spectra demonstrate typical absorption peaks of ZnO at wavelengths ranging from 340 to 360 nm which can be assigned to the intrinsic band-gap absorption of ZnO owing to the electron transitions from the valence band to the conduction band (O_2p_→Zn_3d_) [34]. Reduction in intensity and a noticeable red shift in the absorption edge were observed for the ZnO-NPs samples synthesized at different reaction times. These changes can be attributed to differences in the morphology, particle size and surface nanostructures [34] of the prepared nanoparticles. In addition, the band-gap energies of the products from a plot of (ahv)^2^ versus the photo energy (hv) was consistent with the Kubelka-Munk model [35], which is presented in Figure 4B. The hv value for the ZnO-NPs formed at 12, 24 and 36 h, were 3.39, 3.30 and 3.18 eV, respectively. The band gap energy was increased with decreasing size of nanoparticles from ZnO/T1 to ZnO/T3, as shown by the results of XRD and TEM. The results of this study are also consistent with the previous report which described that the larger band gap of the ZnO-NP with smaller size is due to the quantum confinement effect [36]. In addition the presence of oxygen vacancies in ZnO lattice strucure is another parameter which affects the band gap. It was reported that the increasing of oxygen vacancies results in a narrowing bandgap [37].

#### 2.1.4. Photoluminescence (PL) Analysis

**Figure 5:**
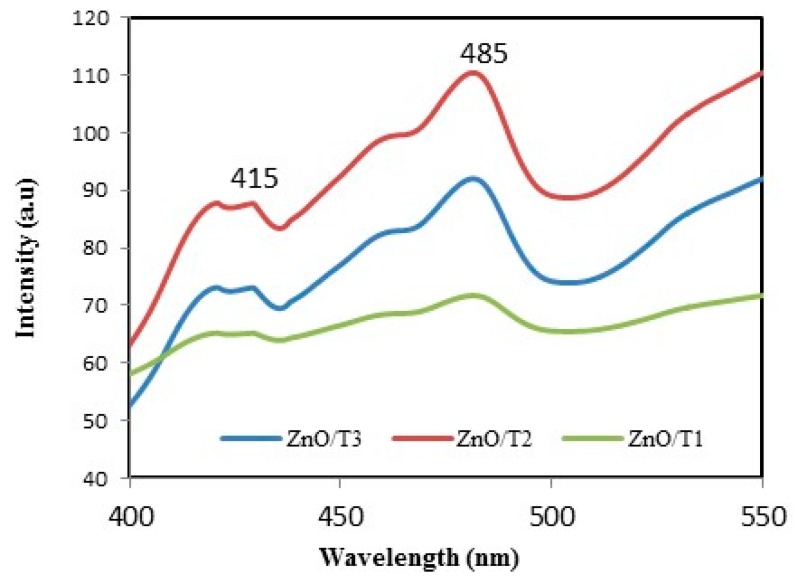
Photoluminescence spectra of ZnO-NPs synthesized at different reaction times.

ZnO luminesces in the visible and UV regions. In the visible region the emission is ascribed to intrinsic defects induced during the synthesis itself. The UV emission is owing to excitonic recombination [38]. Figure 5 shows the PL spectra of biosynthesized ZnO-NPs. It can be seen that the spectra present a wide emission band at wavelengths ranging from 400 to 500 nm. The peak at 415 nm can be ascribed to the V_Zn_ energy level. The emission band located at the wavelength of about 485 nm can be assigned to the electronic transition from the donor level of Zn_i_ to the acceptor energy level of V_Zn_ [39,40]. The alterations in the PL intensities are associated to the recombination rate between the photogenerated electrons and holes. The low PL intensity can be explained as a lower recombination rate between the photogenerated electrons and holes. In other words, a lower recombination rate indicates a higher defect concentration in structure [41]. As observed in Figure 5, ZnO/T2 sample possesses the lowest defect concentration.

#### 2.1.5. Zeta Potential

**Figure 6:**
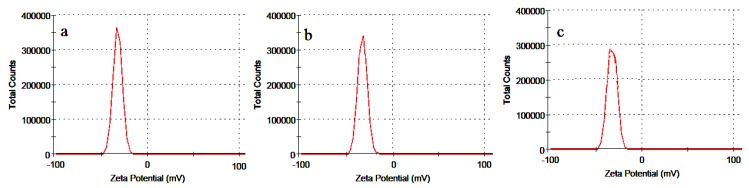
Zeta potential of the (**a**) ZnO/T1; (**b**) ZnO/T2 and (**c**) ZnO/T3 nanoparticles.

The zeta potential values of as-synthesized nanoparticle are shown in Figure 6. The charge greatly influences the distribution of particle as well as the cellular uptake in biological systems. High electric charge on surface of nanoparticles is indicated by elevated absolute zeta potential pointing to large repellent forces in the particles that lead to the stabilizing of the NPs in the medium.

The measured zeta potential values of ZnO-NPs were −32.7, −34.5 and −35.2 mV for ZnO/T1, ZnO/T2 and ZnO/T3, respectively. The negative charge indicates that the particles were well separated and there was a repulsive force between the particles. Thus, the particles did not undergo coalescence to avoid accumulation which ensure long term stability of the particles. The greater magnitude of zeta potential value indicates strong repulsive force between the particles that enhance their stability. Thus, it can be claimed that the synthesized nanoparticles were highly stable, which is the important characteristic of nanoparticle for use in therapeutic purposes.

#### 2.1.6. FTIR Analysis

**Figure 7:**
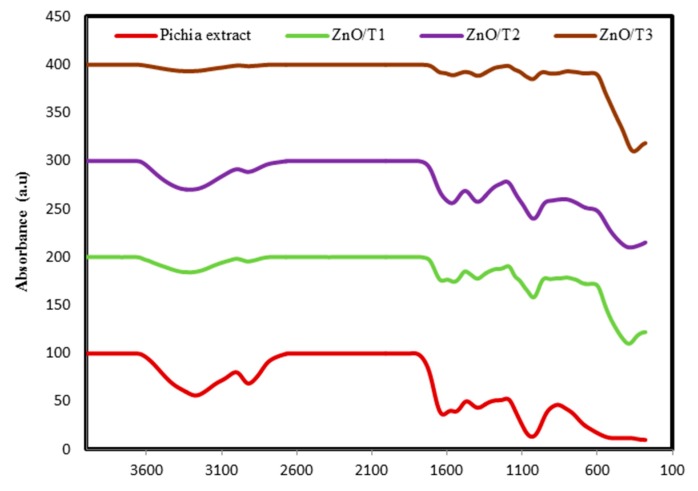
FTIR spectra of *P. kudriavzevii* extract and the synthesized ZnO-NPs.

The FTIR spectra were recorded for the *P. kudriavzevii* extract and the all synthesized ZnO-NPs and are shown in Figure 7. The spectrum of *P. kudriavzevii* extract showed bands at 3345, 1715, 1651, 1456, and 1095 cm^−1^. The wide peak at 3345 cm^−1^ corresponds to strong stretching vibrations of hydroxyl functional groups. The bands at 1715 and 1651 cm^−1^ relate to the carbonyl (C=O) and N-H stretching vibrations in the amide linkages of proteins present in the *P. kudriavzevii* extract. The peak at 1095 cm^−1^ is ascribed to C-OH stretching vibrations. The spectra of the synthesized ZnO-NP samples showed distinct peaks at 3398, 1643, 1442, and 1048 cm^−1^. Upon comparison of the resultant spectra of *P. kudriavzevii* extract and synthesized ZnO-NPs a major reduction in the intensity and shift of the hydroxyl functional group band was observed in the 3345 to 3398 cm^−1^ region and slight reduction and shift changes were also seen in all the peak intensities and positions, indicating the participation of these functional groups in formation and coating of ZnO nanoparticles. From the FTIR results we have concluded that the protein present in the cell free extract could be responsible for the formation as well as capping of ZnO nanoparticles. The absorption bands in the range of 380–340 cm^−1^ in the spectra of biosynthesized nanoparticles ascribed to the Zn-O. The FTIR results clearly show that the surface structure of the ZnO synthesized in different reaction times are different, and this affects the surface layer of Zn-O bonds. This might influence the properties of ZnO-NPs that depend predominantly on the surface structures.

#### 2.1.7. Amount of Zinc Analyzed by ICP-AES

**Figure 8:**
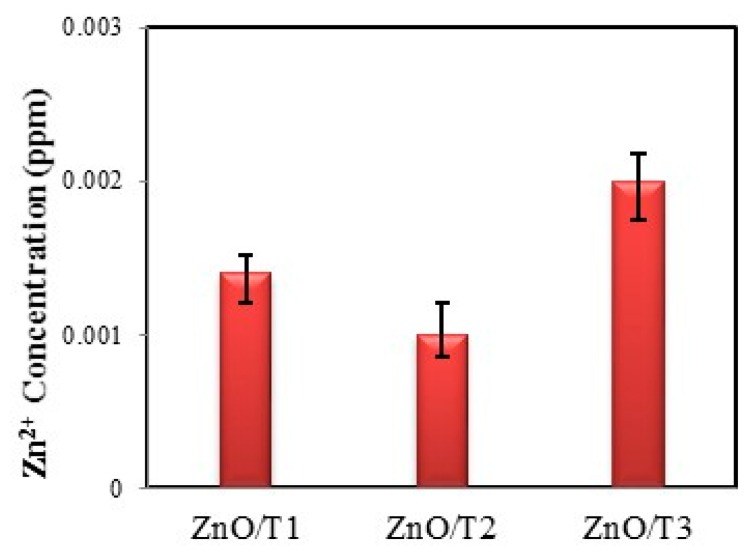
ICP-AES of ZnO/T1; ZnO/T2 and ZnO/T3 nanoparticles.

ICP-AES was used to analyze the amount of zinc in the supernatants of the dispersed ZnO nanoparticles at PH = 7.6. As was shown in Figure 8, the amounts of zinc were small, in the range of (0.001–0.0025 ppm), indicating that the ZnO solutions are relatively free of zinc ions.

#### 2.1.8. Cytotoxicity Studies

**Figure 9:**
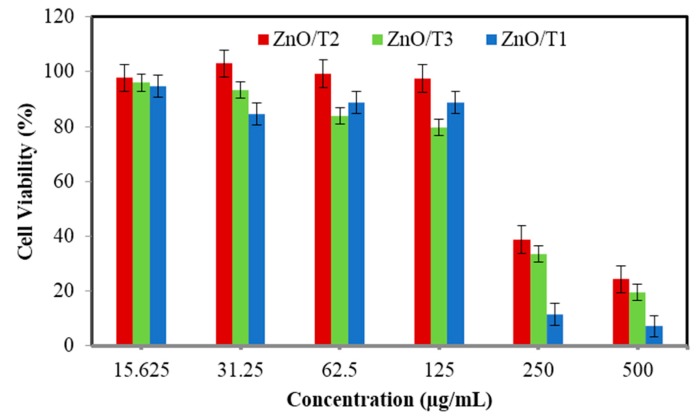
Cytotoxic effect of ZnO-NPs samples on the growth of Vero cells.

The cytotoxicity of the ZnO-NPs was studied in Vero cells. The cell viability was determined by MTT assay after 72 h of incubation at different concentrations (Figure 9). It can be suggested that the ZnO-NPs are low toxic and biocompatible.

ZnO nanoparticles showed a concentration-dependent cytotoxicity. The cell viability decreased with the increase in ZnO concentration. The IC_50_ value for ZnO/T1, ZnO/T2 and ZnO/T3 were 190, 215, and 238 µg/mL, respectively. The lower IC_50_ value indicated that ZnO/T1 has higher cytotoxicity which can be ascribed to greater cellular uptake and therefore increased the intracellular ZnO concentration. It is documented that a diversity of parameters, for example a nanoparticle’s shape, size, surface charge and dissolution of nanoparticles affect the cytotoxicity of nanoparticles [42]. As the surface charge of the all synthesized ZnO-NPs is relatively similar and is in the range of ~32–35 (mV), the effect of surface charge in cellular uptake and consequently their cytotoxicity is negligible. The solubility of oxide NPs, such as ZnO, is another parameter which effects on their cytotoxicity in mammalian cell lines [43]. Slow dissolution of ZnO-NPs in an aqueous medium at pH 7.6 has been demonstrated [44]. As the all experimental tests have been carried out in an aqueous medium at pH 7.6, and based on the ICP results the solutions are relatively free from Zn ions, so an effect of dissolution of ZnO is less probable. It seems that an influence of size and morphology on the cytotoxicity of synthesized ZnO-NPs is more probable. However, the exact mechanism and role of size and morphology of the nanoparticle in cellular uptake requires detailed investigation. One of the possible reasons may be due to easily penetration of small size nanoparticles in cell walls, which increases the intracellular concentration as compared to larger sizes and agglomerated nanoparticles [45].

#### 2.1.9. DPPH Radical Scavenging Activity

DPPH is a stable free radical. It can easily accept hydrogen from a corresponding donor to become a stable diamagnetic molecule. The odd electron of nitrogen atom in DPPH is reduced by receiving a hydrogen atom from antioxidants to the corresponding hydrazine [46], and its solutions lose the characteristic of deep purple color (λ_max_ 515–517 nm).

**Figure 10:**
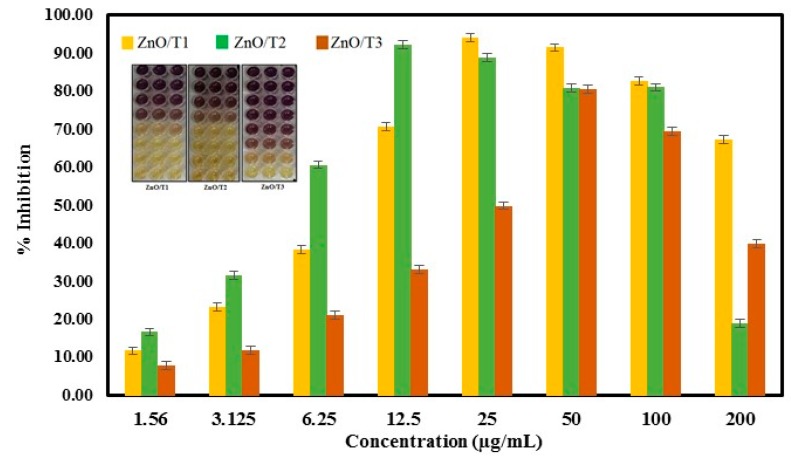
Scavenging capacity of prepared ZnO-NPs, (Inset) color change of DDPH after adding ZnO-NPs.

The reducing activity of ZnO-NPs samples was measured spectrophotometrically by measuring the DPPH color change from purple to yellow (inset of Figure 10). The percentage of DPPH radical scavenging activity is shown in Figure 9. The IC_50_ values of DPPH radical scavenging activities were 10 ± 0.52, 5.26 ± 0.42 and 25.46 ± 0.35 µg/mL for ZnO/T1, ZnO/T2 and ZnO/T3, respectively. The results obtained in the DPPH assay suggested effective free radical inhibition by the ZnO-NPs samples. The antioxidant efficacy of green synthesized ZnO-NP samples can be related to the phytochemicals bounded to the surface of nanoparticles. As was shown in the FTIR results, the ZnO-NPs synthesized in different incubation times have different surface modifications, which affect their antioxidant activities. The ZnO/T2 and ZnO/T1 with a large amount of phytochemicals bounded to their surfaces showed the highest free radical scavenging activities, respectively, compared to ZnO/T3. In addition, the activity depends on the site of attachment of the metals and its consequent impact on the activity of the antioxidant agent [47,48].

It was found that the DPPH radical scavenging activity of the ZnO-NPs decreased with increasing doses, because of the reduced solubility of ZnO-NPs and inadequate DPPH content at higher concentration. Similar results have been reported in a previous study [49]. The maximum scavenging efficacy (%) was found to be 94.55% in 25 µg/mL, 92.37% in 12.5 µg/mL and 82.42% in 50 µg/mL for ZnO/T1, ZnO/T2 and ZnO/T3 samples, respectively.

#### 2.1.10. Antimicrobial Effect of ZnO-NPs

The antimicrobial activity of the bioformed ZnO-NPs was investigated against a variety of Gram-positive and negative pathogenic bacteria by the disk diffusion approach. *B. subtilis. S. aureus*, *S. epidermidis*, *E. coli* and *S. marcescens* were inhibited via biosynthesized ZnO-NPs. The antibacterial activities of the samples varied depending upon the microorganism and the highest inhibition zone was observed for *S. epidermidis*, while the ZnO-NPs were not significantly effective against other Gram positive and negative bacteria (Table 1). The difference in efficiency can be ascribed to the differences in the structural and chemical composition of the cell wall of the various types of micro-organisms tested in this study [50]. It was documented that the cell membranes of Gram-negative bacteria such as *E. coli* and *S. marcescens* tested in this study, have an external lipopolysaccharide (LPS) membrane that protects the peptidoglycan layer. Furthermore, it helps bacteria to survive in environments where exterior materials exist that can damage it [51]. On the other hand, the Gram-positive bacteria such as *S. aureus* and *B. subtilis* have some strategies to survival under harsh conditions. One of the important strategy is the formation of protective endospores to tolerate extreme environmental conditions [52]. It is worth noting *S. aureus*, like *methicillin-resistant S. aureus* (MRSA) is resistant to many antibiotics [53].

**Scheme 1:**
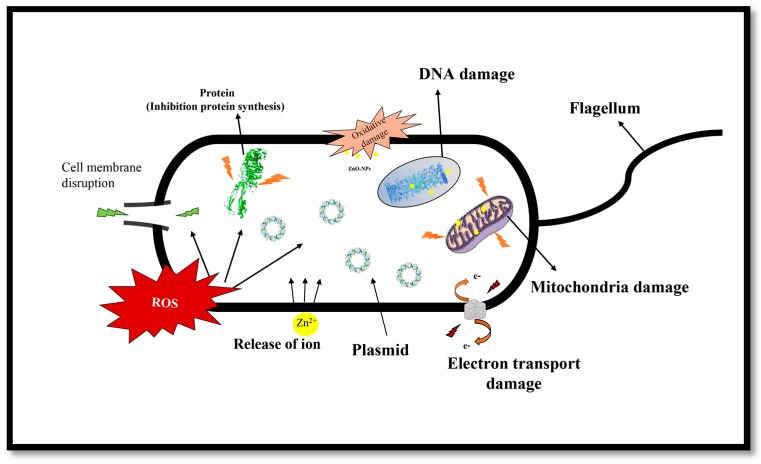
Microbial cell damage through ROS generation and release of zinc ions mechanisms.

It was reported that ZnO-NPs displayed bactericidal effects on Gram-negative and Gram-positive bacteria, as well as spores resistant to high pressure and temperature [54]. Enhancement of the surface area in the NPs was considered responsible for the improvement of the antibacterial properties of ZnO nanoparticles in comparison with microparticles [55,56,57]. A good understanding of the antimicrobial mechanisms of ZnO-NPs is not yet elaborated and reported. Zhang et al. proposed that the generation of hydrogen peroxide is possibly the key process behind the antibacterial property. There is also a suggestion that the antibacterial effect of ZnO could be due to the accumulation of ZnO particles on the bacterial cell surface [58]. Furthermore, release of zinc ions, generation of ROS (reactive oxygen species) (Scheme 1) on the surface of the particle and internalization of nanoparticles, may also be regarded as probable causes of cell damage. Transmembrane interruption and electron transportation in some metal nanoparticles e.g., (Ag) and (Zn) can also be added to the list [59,60].

ZnO appears to react differently under different pH conditions, exhibiting excellent stability above pH 7.0, but much greater dissolution at lower pH [61]. As the all antimicrobial tests performed in the medium at pH 7.6, and all tested solution samples were relatively free from Zn ions (based on the ICP results), the effect of dissolution of ZnO and Zn release to explain the antimicrobial activity of ZnO-NPs is less probable. Thus, other factors such as generation of active molecules or accumulation of ZnO particles on the bacterial cell could be more probable reasons of cell damage.

**Figure 11:**
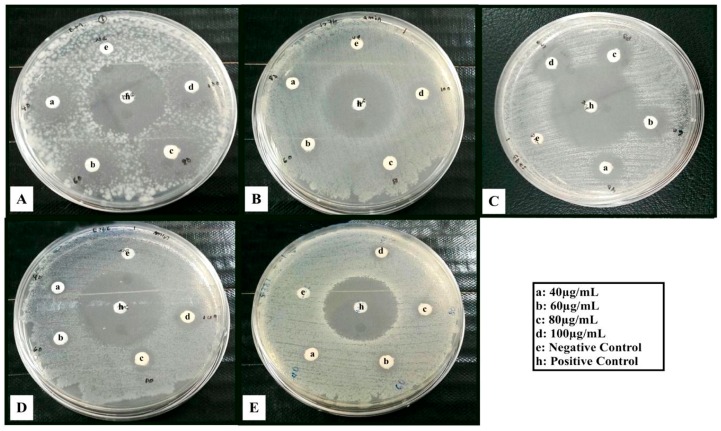
Inhibition zones (mm) of ZnO/T2 against (**A**) *S. epidermidis*; (**B**) *B. subtilis*; (**C**) *S. aurous*; (**D**) *E. coli* and (**E**) *S. marcescens* bacteria.

According to the results of this study, ZnO/T2 was more effective on all pathogenic bacteria so far tested (Figure 11, Table 1) as compared to ZnO/T1 and ZnO/T3; these results can be explained by the morphology and size of these nanoparticles. It was documented that the antimicrobial activity of nanoparticles is influenced by their morphological and physicochemical characteristics [56,62]. We showed that the various concentrations (40, 60, 80 and 100 µg/mL) of ZnO-NPs have antimicrobial activity and this effect can be enhanced by increasing the concentration of NPs. For *S. epidermidis* in ZnO/T2, showed the inhibition zone was increased from 8 ± 0.026 mm to 19 ± 0.32 mm when the concentration of NPs was increased from 40 µg/mL to 100 µg/mL. These results are in agreement with the previous studies which have shown that the antibacterial activity of ZnO nanoparticles is dependent upon the nanoparticle concentrations [63]. In this study, the highest concentration (100 µg/mL) of ZnO/T2 against *S. epidermidis* was a major contribution to antimicrobial activity. In a similar study, Taylor and colleagues [64] reported that the inhibition for *S. epidermidis* pathogen treated with super-magnetic iron oxide nanoparticles was also concentration-dependent. The effects of concentration and particle size on antibacterial activity of ZnO-NPs have been reported [65]. They claimed that the antibacterial effects of ZnO-NPs were significantly enhanced with increasing concentration and decreasing size of particles.

## 3. Materials and Methods

### 3.1. Materials

Potato dextrose agar (PDA), potato dextrose broth (PDB), zinc acetate dihydrate (Zn(Ac)_2_·2H_2_O) were procured from Sigma Aldrich (Kuala Lumpur, Malaysia). The mango fruits were purchased from a local shop market in Selangor (Kuala Lumpur, Malaysia).

### 3.2. Isolation of Pichia Kudriavzevii

**Figure 12:**
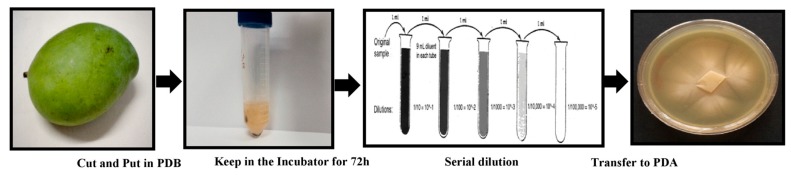
Yeast isolation process.

The whole fruits were kept at room temperature until rotten. The isolation of yeast was done by standard serial dilution methods as shown in Figure 12. Briefly, about 1 g of fruit was cut into small pieces (3 mm diameter) and put into 9 mL of potato dextrose broth (PDB) and then kept in an incubator for 72 h at 30 °C. After that, the sample was diluted up to 10^−4^, 10^−6^ and 10^−8^ using distilled water and finally plated onto potato dextrose agar (PDA) with 100 μg/mL ampicillin as an antimicrobial agent. The yeast was grown on potato dextrose broth (PDB) after incubation at 28 °C in vibrating incubator at 150 rpm for 72 h. Fungal mycelia were separated from the culture medium through centrifugation (10,000 rpm,10 min, and 4 °C) and washed several times with sterile water in order to remove any components of the medium. Typically, 20 g of biomass was re-suspended in 100 mL of sterile deionized Milli-Q water and further incubated under the above described conditions for 72 h. After incubation, biomass was separated by filtration using Whatman filter paper No. 1, and the fungal cell-free filtrate containing extracellular secretions was collected to use for synthesizing of nanoparticles.

### 3.3. Isolated Yeast Molecular Identification

Genomic DNA of the variety was extracted using the method described by Rainey et al. [66]. Primers ITS1 (5′-TCCGTAGGTGAACCTGCGG-3′) and ITS4 (5′-TCCTCCGCTTATTGATATGC-3′) were used by PCR to amplify 18s rRNA gene which was separated on 1% agarose gel.

**Figure 13:**
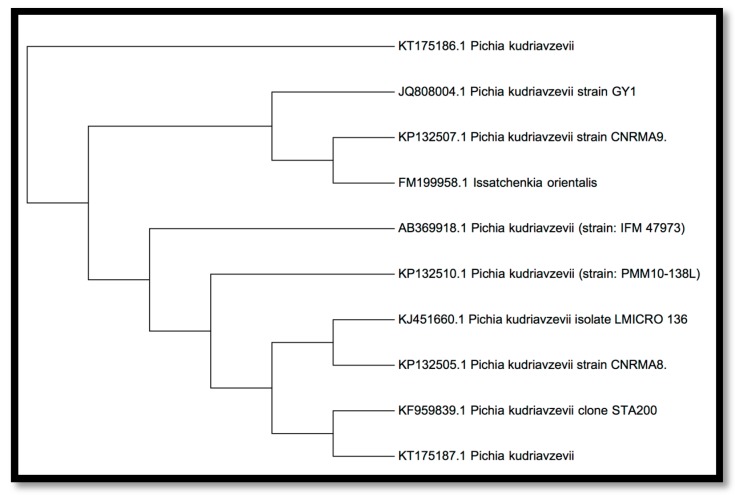
Phylogenetic tree showing relationship of JQ808004 with other strains of *Pichia* species based on 18s rRNA gene sequences retrieved from NCBI GenBank.

A nucleotide sequence database search was used for evaluating the phylogenetic state of the isolated type (*Pichia kudriavzevii*). The NCBI GenBank BLAST program was used for this purpose. Having retrieved sequence data for related from NCBI GenBank, the results of the nucleotide sequencing were lodged with the GenBank National Centre for Biotechnology information (NCBI) under accession number JQ808004. The untainted colonies were acquired and the isolation was recognized as *P. kudriavzevii* based on the molecular characterization via 18s rRNA sequencing, moreover it has 521 bp (Figure 13).

### 3.4. Extracellular Synthesis of ZnO-NPs

ZnO-NPs were synthesized using 100 mL of fungal cell-free filtrate mixed with 10 mL zinc acetate dihydrate (Zn(Ac)_2_·2H_2_O) solution (10 mmol/L) in a 250 mL Erlenmeyer flask incubated at 35 °C in the shaking incubator, agitated at 150 rpm for 12, 24 and 36 h. After the color of the solutions changed from light brown to pale and deep white, the biomass products were collected by centrifugation at 10,000 rpm for 10 min and then dried at 150 °C for 6 h. The samples obtained at the different times of incubation (12, 24 and 36 h) were labelled as ZnO/T1 ZnO/T2 and ZnO/T3 respectively. The recovered filtrate was stored at 5 °C in borosilicate flasks until use.

### 3.5. Characterization of ZnO-NPs

UV-Vis spectra of ZnO-NPs powders were measured using a Lambda 25 spectrophotometer (Perkin Elmer, Waltham, MA, USA) at wavelengths ranging from 200 to 800 nm. The X-ray diffraction data were recorded by PXRD (Philips, X’pert, Almelo, The Netherlands) at 40 kV and 30 mA from 2θ = 10° to 80° with nickel-filtered Cu (λ = 1.542 Å) at room temperature. The morphology and size of ZnO-NP samples were examined by using a Transmission Electron Microscope (TEM, Hitachi H-700, Tokyo, Japan) in 120 kV. The particle electrostatic charge was evaluated using the laser doppler electrophoresis technique, whereby 100 µL of the solution was diluted in 1.5 mL of water. Subsequently, it was poured into a Zetasizer-nano instrument cuvette (Malvern, UK); the results are stated as zeta potential (ZP). Photoluminescence (PL) spectra were recorded using a spectrophotometer (LS 55, Perkin–Elmer, Beaconsfield, UK) with an excitation wavelength of 320 nm.

### 3.6. Inductively Coupled Plasma-Atomic Emission Spectroscopy (ICP-AES)

For in vitro studies, stock suspensions of ZnO-NPs of the different samples were prepared in loading media (pH 7.6), and stored for 3 days at 22 °C. The released zinc ions were eliminated before the biological analysis by ultracentrifugation 30 min at 20,000× *g*. Then 10 mL of the supernatant was transferred into a test tube that contained 0.5 mL of concentrated nitric acid (HNO_3_). The solution subjected to zinc analysis by inductively coupled plasma atomic emission spectrometry (ICP-AES) (Perkin Elmer Plasma 1000).

### 3.7. Free Redical Scavenging Activity

1-Diphenyl-2-picrylhydrazyl (DPPH) radical assay ZnO-NPs samples were evaluated for the scavenging effect on DPPH radical according to the method of Blois [67]. Different concentrations (1.56, 3.125, 6.25, 12.5, 25, 50, 100 and 200 µg/mL) of ZnO-NPs samples were added, in equal volume, to 0.1 mM methanolic DPPH solution. The reaction mixture was incubated for 30 min at ambient temperature under shaking condition and the absorbance was read at 517 nm. The butyl hydroxyl toluene (BHT) was used as positive control. All tests were carried out in triplicate. The DPPH radical scavenging activity (RSA) was expressed in percentage of inhibition using the following formula:

$$\%\;\mathrm{RSA}=[\frac{A_{\mathrm{control}}-A_{\mathrm{sample}}}{A_{\mathrm{control}}}]\times 100$$

where *A*_control_ is the absorbance of the blank control and *A*_sample_ is the absorbance of the test sample.

### 3.8. Cytotoxicity Studies

Cellular toxicity of ZnO-NPs was evaluated by MTT assay in Vero cells. Vero cells are one of the most common mammalian continuous cell lines used in research. These cells are usually used in assessment of the effects of chemicals, toxins and other substances on mammalian cells at the molecular level. About 10,000 cells per well were cultured in 96 wells plate and allowed to attach overnight. After one day, cells were incubated with different concentrations of ZnO-NPs, and untreated cells were used as control. Cells were incubated in CO_2_ incubator at 37 °C, pH 7.6, for 48 h. The solution of each well was replaced with fresh serum free media and 10 µL of MTT reagent (5 mg/mL) was added. After 4 h, the media was removed, 200 µL DMSO was added and incubated for 20 min. The optical density was then measured at 570 nm using an enzyme-linked immunosorbent assay universal microplate reader (Bio Tek Instruments, Inc., Winooski, VT, USA). The cell viability was determined as a percentage based on the absorbance measured relative to the absorbance of untreated or control cells. The half maximum inhibitory concentration (IC_50_) values were determined by non-regression analysis using GraphPad Prism v. 5.03 (GraphPad Software Inc., La Jolla, CA, USA).

### 3.9. Antimicrobial Screening of ZnO-NPs

The antibacterial activity of zinc oxide nanoparticles was evaluated against Gram-positive (*Bacillus subtilis B*29, *Staphylococcus aureus S*276, *Staphylococcus epidermidis S*273) and Gram-negative (*Escherichia coli E*266, *Serratia marcescens S*381) bacteria by using a disk diffusion test. All bacterial cultures were grown in Muller-Hinton Agar, pH 7.6. The bacterial inoculum was standardized to 0.5 MF units, meaning that approximately 1.5 × 10^8^ colony-forming units of each bacterium were inoculated onto a plate. The sample solution with different concentrations (40, 60, 80, 100 µg mL^−1^) were added to the top of the disks and inoculated with the cultures. Streptomycin was used as a positive control. After incubation at 30 °C for 24 h, the zones of inhibition were measured.

## 4. Conclusions

For the first time, the potential use of a newly isolated yeast strain (*P. kudriavzevii*) to synthesize ZnO-NPs using a green method is demonstrated in this study. The ZnO nanoparticles were found to have the hexagonal wurtzite structure with an average crystallite size of ~10–61 nm. The reaction duration was found to play a critical role in the size, shape and distribution of ZnO-NPs. The as-synthesized nanoparticles were less toxic and displayed antioxidant and antibacterial activities. Biosynthesized ZnO-NPs showed a strong DPPH radical effect with a dose dependent activity. The synthesized nanoparticles also displayed antibacterial activity against both Gram-positive (*S. aureus*, *B. subtilis*, *S. epidermidis*) and Gram-negative bacteria (*E. coli* and *S. marcescens*). The ZnO-NPs were noticeably effective on *S. epidermidis* pathogen. In conclusion, this procedure may be suitable for preparing nanomedicines and targeted drug delivery in near future.

## Figures and Tables

**Table 1:** Inhibition zones (mm) of ZnO-NPs by disk diffusion method against different Gram-negative and Gram-positive bacteria.

Sample	Concentration µg/mL	*S. epidermidis* (A)	*B. subtilis* (B)	*S. aureus* (C)	*E. coli* (D)	*S. marcescens* (E)
**ZnO/T1**	40	7 ± 0.24	5 ± 0.11	6 ± 0.38	8 ± 0.42	5 ± 0.21
	60	9 ± 0.27	6 ± 0.23	6 ± 0.25	7 ± 0.24	5.6 ± 0.16
	80	11 ± 0.35	7 ± 0.15	7 ± 0.32	8 ± 021	6 ± 0.11
	100	14 ± 0.25	7 ± 0.41	9 ± 0.24	9 ± 0.21	7 ± 0.21
**ZnO/T2**	40	8 ± 0.26	6 ± 0.21	7 ± 0.28	9 ± 0.32	6 ± 0.18
	60	12 ± 0.37	7 ± 0.28	7 ± 0.30	8 ± 0.33	6.5 ± 0.26
	80	15 ± 0.25	8 ± 0.26	8 ± 0.42	9 ± 0.31	7 ± 0.21
	100	19 ± 0.32	9 ± 0.32	10 ± 0.36	10 ± 0.22	9 ± 0.24
**ZnO/T3**	40	8 ± 0.16	6 ± 0.29	6 ± 033	9 ± 0.12	6.4 ± 0.14
	60	11 ± 0.32	7 ± 0.58	7 ± 0.38	7 ± 0.27	6.1 ± 0.23
	80	13 ± 0.21	7 ± 0.24	7 ± 0.30	8 ± 0.51	7.2 ± 0.20
	100	16 ± 0.37	8 ± 0.22	9 ± 0.46	9 ± 0.21	8.5 ± 0.31
